# Neural Processing of Sexist Comments: Associations between Perceptions of Sexism and Prefrontal Activity

**DOI:** 10.3390/brainsci13040529

**Published:** 2023-03-23

**Authors:** Michelle Jin Yee Neoh, Andrea Bizzego, Jia Hui Teng, Giulio Gabrieli, Gianluca Esposito

**Affiliations:** 1 Psychology Program, School of Social Sciences, Nanyang Technological University, Singapore 639818, Singapore; 2 Department of Psychology and Cognitive Science, University of Trento, 38068 Rovereto, Italy; 3 Neuroscience and Behaviour Laboratory, Italian Institute of Technology, 00161 Rome, Italy

**Keywords:** perceived sexism, gender stereotypes, neuroimaging

## Abstract

Sexism is a widespread form of gender discrimination which includes remarks based on gender stereotypes. However, little is known about the neural basis underlying the experience of sexist-related comments and how perceptions of sexism are related to these neural processes. The present study investigated whether perceptions of sexism influence neural processing of receiving sexist-related comments. Participants (*N* = 67) read experimental vignettes describing scenarios of comments involving gender stereotypes while near-infrared spectroscopy recordings were made to measure the hemodynamic changes in the prefrontal cortex. Results found a significant correlation between participants’ perceptions of sexism and brain activation in a brain cluster including the right dorsolateral prefrontal cortex and inferior frontal gyrus. There was a significant gender difference where female participants showed a stronger negative correlation compared to male participants. Future research can expand on these initial findings by looking at subcortical structures involved in emotional processing and gender stereotype application as well as examining cultural differences in perceptions of gender stereotypes and sexism.

## 1. Introduction

Women experience sexism across a multitude of contexts in their everyday lives including the workplace and schools where a number of studies have highlighted the prevalence of sexist incidents experienced in daily life (e.g., [1,2]). Sexist incidents include being the target of traditional gender-role stereotypes, sexual objectification and degrading comments (see [3]). Sexist elements can be infused in the comments and judgments we make about others, including unsolicited and gratuitous sexist remarks which often target appearances, abilities and traits. Such sexist remarks, which can be derogatory and belittling, can be considered to be a form of criticism. According to ambivalent sexism theory [4,5], hostile sexism consists of overtly negative attitudes, stereotypes and behaviour towards women where hostile sexist comments convey antipathy and antagonistic attitudes toward women. Such hostile sexist comments are more readily identified and perceived to be more distressing and sexist [4,6]. This suggests the possible interplay of an individual’s perceptions of sexism in moderating their response towards receiving sexist remarks directed at them. Hence, the present study examines whether perceptions of sexism influence the response and neural processing involved in sexist-related comments.

### 1.1. Neural Processing of Sexist Events

Negative emotions such as feelings of anger, upset, weakness and sadness are commonly reported reactions to experiences of sexism [3,7,8,9,10], similar to negative emotional reactions in response to criticism. In a similar vein, neuroimaging studies examining the neural correlates of criticism have proposed the involvement of three processes; (i) emotion reaction, (ii) emotion regulation and (iii) social cognitive processing [11]. Hence, given the similarity in responses to experiences of sexism and criticism, this suggests that neural processes underlying emotional reactivity and emotion regulation processes are also involved in the experience of sexism. Negative emotional reactions have been linked to activities in areas such as the prefrontal regions of the rostral anterior cingulate cortex [12,13,14] and subcortical-limbic regions [15,16]. Increased activity in the dorsolateral prefrontal cortex (dlPFC) is observed in emotion regulation [17,18,19,20]. Hence, activation in prefrontal areas involved in emotional reactivity and regulation can be expected in response to experiences of sexist events.

A large number of studies has investigated gender differences in neural activation involved in emotional reactivity and emotion regulation in terms of the levels of brain activation as well as the brain regions involved. A meta-analysis found that the right amygdala, dorsomedial PFC and left insula were activated in both sexes for negative emotions where increased brain activation in response to negatively-valenced stimuli was more likely to be observed in subcortical and limbic areas—specifically, the left amygdala and the medial prefrontal cortex (mPFC)—for females [21]. With regards to emotional reactivity, females have been found to be more reactive to emotional stimuli, specifically unpleasant, threatening or traumatic stimuli [22,23,24]. For example, higher activity was observed in the amygdala, prefrontal and temporal cortices in females in response to negative picture stimuli compared to males [25]. In a study by [26], significant levels of activation were observed in the bilateral caudate nuclei and left putamen in response to unpleasant words relating to interpersonal relationships compared to neutral words only in the female group but not the male group. The levels of activation were also found to be negatively correlated with the ratings of pleasantness of the words concerning interpersonal conflict. Similarly, ref. [27] found gender differences in brain activation during the processing of negatively-valenced words compared to non-words in regions where higher activation was observed in the perirhinal cortex and hippocampus in females but higher activation was observed in the right supramarginal gyrus in males. Gender differences in emotional speech processing were found where a significantly higher activation in the left IFG was observed when emotional prosody and word valence was incongruous, suggesting gender differences in semantic processing [28]. With regards to emotion regulation, results from studies on explicit emotion regulation have not been consistent (see [29]). A study found a smaller increase in prefrontal activation and greater decrease in amygdala activation in males compared to females during a cognitive reappraisal task although comparable decreases in self-reports of negative emotion were observed in both groups [30]. However, ref. [25] found lower activation in parts of the OFC, anterior cingulate and dlPFC in females compared to males in a similar task, but no significant gender differences in amygdala activity were observed. In one study where general instructions to downregulate negative emotion in response to emotional stimuli were given, both males and females showed higher activation in the left anterior cingulate gyrus but males showed greater activation in the left dlPFC, lateral orbitofrontal gyrus and right anterior cingulate gyrus whereas females only showed greater activation in the left medial orbitofrontal gyrus [31]. Hence, we expect that there may be gender differences in neural activation in areas involved in emotion reactivity and regulation in response to sexist-related comments.

### 1.2. Neural Processes of Sexism and Gender-Related Stereotypes

Although neuroimaging studies have not been conducted on experiences of sexism specifically, we can draw on findings from other relevant fields of study such as prejudice, experiences of discrimination and stereotyping in studying the neural processes involved in experiences of sexist events, since sexism is a form of discrimination based on gender. While little is known about neural correlates involved in sexist-related comments involving gender stereotypes, a significant number of studies have investigated gender stereotype processing. Neuroscientific research into the neural basis of prejudice and stereotypes suggests the interactions of different networks of neural structures (see [32]). Key brain areas involved in the neural basis of prejudice include the amygdala, insula, ventral medial PFC and OFC. Activity in areas associated with evaluative processing such as the ventromedial PFC and amygdala and representation of action knowledge such as the supramarginal gyrus and middle temporal gyrus was found to underpin stereotyping processes [33]. In terms of cortical structures, the orbital frontal cortex (OFC) has been proposed to be involved in complex evaluations of people based on group membership and regulation of social behaviour [34,35]. The OFC processes affective cues [36] and supports the monitoring of social cues and behavioural adjustments [34], suggesting that it could be involved in processing sexist-related comments, which involves information on group membership and affective responses.

Experiences of discrimination and stereotyping also involve social cognition about the source of discriminatory behaviour. One brain area that has been found to be involved in social cognition and processing social information is the medial PFC (mPFC) [37,38,39]. The mPFC has been suggested to be a part of a social-cognition network including the temporo-parietal junction, superior temporal sulcus and amygdala, which supports core aspects of person construal [37,40,41,42]. Neuroimaging evidence indicate that making social judgments about people such as impression formation of a person (e.g., [43,44]), interpersonal affect (e.g., [45,46]) and theory of mind (e.g., [47,48,49]) have been associated with observations of differential mPFC activation. The mPFC has also been suggested to play a prominent role in stereotyping. The mPFC has been shown to be involved in the representation of other-referential social information—in particular, outgroup stereotyping [50], which is relevant in the context of sexism. For example, activity in the mPFC has been linked to gender-related automatic beliefs and stereotyping during behavioural tasks such as the implicit association test [51,52]. The ventromedial and dorsomedial PFC sectors are also involved in modulating gender stereotypical associations. The ventromedial PFC has been linked to emotional processing, self-knowledge, “mentalizing” and the perception and judgment of others (see [53]). Ventromedial PFC activity was greater for stereotype-compatible judgments compared to neutral judgments and the ventromedial PFC activation was also found to correlate with the strength of explicit and implicit gender stereotypes [33]. The findings from these studies suggest that the mPFC contributes to the generation of stereotype-based judgements, suggesting that differential mPFC activation can be expected to be observed towards sexist-related and non-sexist comments involving gender stereotypes.

Self-regulation and cognitive control of social emotions and behaviour such as the inhibition of social stereotypical responses are mainly supported by prefrontal areas. Greater activation in the left dlPFC was observed when responses contrasted with stereotypical gender or racial bias [51] and during inhibitory tasks involving implicit associations [54]. The lateral PFC (regions such as BA44, BA45 and BA47 referred to as the inferior frontal gyrus (IFG)) has been linked to the coordination of control over action and attention including action implementation and inhibition [55,56]. The role of the right IFG in domain-general response inhibition supports the possibility that right IFG activation during stereotype judgment tasks may be reflective of efforts towards the inhibition of the influence of stereotypes on behaviour [57]. On the other hand, greater right IFG activity has been observed during the judgment of the application of gender stereotypical traits to male and female individuals as opposed to trials without stereotype application [58]. Moreover, such a pattern of brain activation was positively correlated with strength of gender stereotypic associations. The authors suggested that the right frontal cortex activity may be related to the application of category knowledge for the purpose of social judgment, where right frontal activity may be related to social cognition involving category-based semantic retrieval as observed in previous studies on categorization [59,60]. In addition, the mPFC is also proposed to support the regulation of behavioural responses in accordance with social cues, where evidence from ERP studies suggest the contribution of mPFC and rostral anterior cingulate cortex (ACC) activity to behavioural control guided by external social cues [37]. Hence, we can expect to observe dlPFC activity involved in sexist-related comments and the extent of activation may be related to the perceptions of the comments as sexist.

Specific gender-oriented stereotypes have been associated with particular words and found to be automatically activated when such words are encountered across different languages such as English, Spanish, Italian and German [61,62,63,64,65]. These psycholinguistic studies showed that words conveying gender stereotype incongruency are processed more slowly than those congruent with gender stereotypes. In this respect, neurophysiological studies on gender stereotypes have provided further evidence indicating differences in processing of gender stereotypes. Two event-related potential (ERP) components, N400 and P600, have been consistently found in response to gender stereotype violations. In terms of the brain areas involved in generating the N400 component towards stereotype violations, ref. [66] found that one of the most active brain areas was the right middle frontal gyrus, an area described as the main neural basis for stereotype representation (see [67,68]). The medial temporal gyrus, which is involved in person representation [69,70], was also implicated in [66]. This observation aligned with previous findings linking gender bias representation to activity in the medial temporal gyrus, superior frontal gyrus and superior temporal gyrus [33]. Essentially, the key brain areas found to be involved in gender stereotype violations were previously associated with prejudice violation, social cognition and person knowledge [66].

Gender differences in the neural correlates of gender stereotyping processes have not been explored in depth as well. In the study by [66], the findings indicated that enhanced N400 and P600 ERPs in response to items violating gender stereotypes were mostly in men. One transcranial magnetic stimulation (TMS) study found that the application of TMS to the left dlPFC and the right anterior dorsomedial PFC resulted in increased gender stereotypical bias in male participants compared to the application of TMS to a control site but not for female participants [71]. Similarly, a transcranial direct current stimulation (tDCS) study found that the application of anodal tDCS to the mPFC led to a significant reduction in the implicit gender stereotypes amongst male participants but not female participants [72]. Hence, the present study aimed to investigate gender differences in the neural correlates of self-referential gender stereotypes and the association with perceptions of sexism in the context of sexist-related comments.

### 1.3. Significance and Aim of the Present Study

While studies have looked at how women respond towards experiencing sexism, neuroimaging studies investigating the neural correlates of responses towards sexist events, specifically sexist-related comments, have not been conducted as far as we are aware. Neuroimaging studies have been conducted on experiences of discrimination such as racial bias and stereotyping in general, but sexism and perceptions of sexist events have not been the focus of these studies despite the prevalence of sexist events in the daily lives of women and the well-established link between experiences of sexism and psychological distress. A majority of previous neuroimaging studies also looked at other-referential gender stereotypes and few studies have looked at self-referential gender stereotypes. Hence, as far as we are aware, this is the first neuroimaging study examining the neural processes involved in experiences of sexist events, specifically sexist-related comments. Examining the relationship between individual perceptions of sexism and neural processes contributes to understanding how the neural responses are associated with the individual’s perceptions and reactions to self-referential, sexist comments, which can help to provide insight into understanding the neural mechanisms underlying the link between experiences of sexism and psychological distress. Hence, the present study aims to investigate the association between perceptions of sexism and neural activation in the prefrontal cortex in response to sexist-related versus non-sexist comments.

There are two hypotheses in the present study. Firstly, in line with previous studies on gender stereotypes indicating the role of the inferior frontal gyrus, dlPFC and mPFC activity, we hypothesise that there will be a significant association between the perceptions of sexism and neural activation in these areas. We expect that individuals who perceive and construe sexist elements in comments made by others to a greater extent would experience a greater negative emotional reaction. As a result, we expect that lower dlPFC activity will be associated with higher perceptions of sexism. Secondly, based on previous studies indicating gender differences in gender stereotypical bias, we expect to observe a significant difference between male and female groups in the association between perceptions of sexism and brain activation patterns.

## 2. Method

### 2.1. Participants

The participants (N = 67, male = 29, female = 38, M_age_ = 22.0, SD_age_ = 2.30) were recruited through (i) a psychology undergraduate course and compensated with course credits and (ii) advertisements and compensated with remuneration. The study was approved by the Psychology Ethics Committee at the Nanyang Technological University (PSY-IRB-2020-007) and written informed consent was obtained from participants before completing the experiment.

### 2.2. Experimental Procedure

#### 2.2.1. Experimental Stimuli

The participants read vignettes describing hypothetical scenarios involving comments regarding commonplace themes of academic ability, empathy, accountability and assertiveness. The comments in the scenarios on academic ability and assertiveness included sexist remarks alluding to prevailing gender stereotypes. For example, one scenario was based on the gender stereotype that women are poor at math and sciences whereas men excel in these subject areas [73,74,75] and the stereotype that language and verbal skills tend to better in women rather than men [76,77,78]. An example of the comment for male participants referred to stereotypical ability in math—”Guys usually excel in Mathematics, but I am disappointed with your poor results.” On the other hand, non-sexist scenarios did not refer to any gender stereotypes in the comment being made—for example, “You showed a lack of empathy for your sister’s emotions. I am disappointed with you”. The order of the four different hypothetical scenarios were always the same for all participants. All vignettes were approximately 120 words long.

Each vignette was presented for 50 s, and the offset of each vignette was followed by a fixation point displayed in the centre of the blank screen. Each fixation point lasted for 10 s after which participants were asked to rate the extent to which they thought the comments being made in the scenario were motivated by sexist beliefs on a 5-point scale.

#### 2.2.2. fNIRS Recording

The experiment was carried out in a dimly lit room, where participants were fitted with a NIRS cap and the NIRS signal was calibrated. During the experiment, participants were presented with the vignettes on a laptop while NIRS recordings were being made.

**Figure 1:**
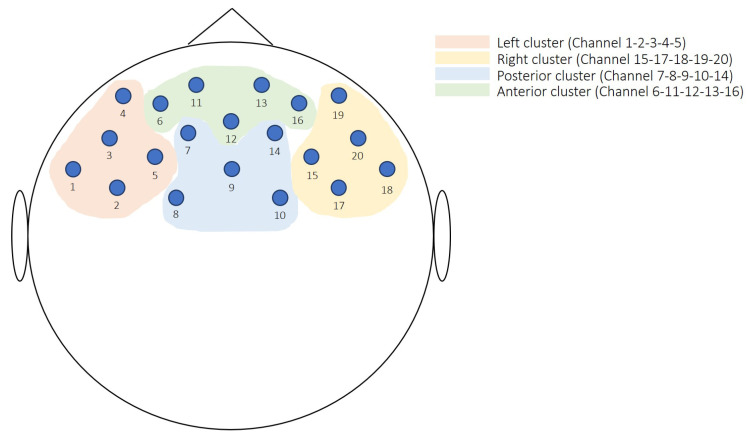
Diagram displaying channel locations and clusters of channels used in the analysis.

The fNIRS recordings were made with the functional NIRS imaging system (NIRSport, NIRx Medical Technologies LLC, Glen Head, NY, USA) which operates using light of wavelength 760–850 nm. This system measures relative oxygenated hemoglobin (HbO) and deoxygenated hemoglobin (Hb) concentrations which indicate cerebral activation and deactivation. fNIRS allows monitoring of local blood oxygenation where greater concentrations of HbO are observed in brain regions with higher activity. fNIRS is based on neurovascular coupling, where cerebral blood flow necessary for brain function is coupled with neuronal activity. Activity in a particular brain region results in increases in cerebral blood flow and increased cerebral oxygenation, which is then detected by the fNIRS (see [79]) for review). The NIRS device consists of LED-sources (emitting optode) that transmit long-wave light to cortical tissues and detectors (receiving optode) that measure the intensity of returning light. The optical signal was recorded at a sample rate of 7.81 Hz. In this study, the hemodynamic changes of the PFC were measured. The configuration of 8 sources and 7 detectors on the NIRS cap formed a 20 multi-distant channel setup where data from cortical measurements were recorded using the NIRStar Software 15.0 (see Figure 1 for channel locations). The distance between sources and detectors did not exceed the optimal interoptode distance of 3 cm. Probes on the NIRS cap were adjusted at the start of the experiment before calibration of signal quality for each participant.

### 2.3. fNIRS Preprocessing

Preliminary signal processing was conducted using custom scripts developed based on the pyphysio package on Python [80]. Data pre-processing included signal quality check, motion artifact correction and conversion of optical density data to HbO and Hb concentration changes using the modified Beer-Lambert law [81]. Signal quality check is conducted to exclude channels with inadequate signal quality from further processing. Signal quality check was carried out using a Convolutional Neural Network (CNN), trained on a dataset of 548 manually labeled signal portions [82]. The signal quality is assessed based on raw data by channel, on 20 s segments that are extracted using moving windows (15 s overlap). A channel’s signal is considered of good quality if at least 90% of the windows are classified as good quality. Motion artifacts were removed using wavelet filtering [83,84]. Wavelet filtering involves decomposing the data into wavelet coefficients where outliers in the distribution of these wavelet coefficients are assumed to reveal the presence of the artifact. Finally, the filtered signals were converted to HbO and Hb concentrations, using the modified Beer-Lambert law [81]. After the signal quality check and preprocessing stage, the final sample used for data analysis consisted of 58 participants (male = 21, female = 37). Channels were grouped into four clusters: (i) left, (ii) right, (iii) anterior and (iv) posterior clusters of the prefrontal cortex for the statistical analyses (Figure 1). Cluster signals were computed by averaging the channel signals; a cluster signal was computed only if at least 3 channels with good quality were available.

### 2.4. Data Analysis Plan

Statistical analyses were conducted using Python (Version 3.9.7). First, the NIRS data were analysed at two levels: within-subject and group-level. A general linear model (GLM) was run for each participant where individual beta coefficients were calculated for each vignette-block on the processed HbO concentrations. Next, the Spearman correlation between the individual-level beta coefficients and the ratings of sexist beliefs was calculated for each subject and cluster. The dataset analysed in this study is available in the open access institutional data repository at the link: https://doi.org/10.21979/N9/C3JRXS (accessed on 18 January 2022).

According to the first hypothesis, a negative correlation between the perceptions of sexism and neural activation in the PFC areas is expected. To verify this hypothesis, a Wilcoxon signed rank test was used to test the computed Spearman correlation coefficients were significantly negative. Finally, a Kruskal-Wallis test was used to test for significant differences in the Spearman correlation coefficients between the male and female groups. The analysis was replicated for each cluster; Bonferroni correction was applied to correct for multiple comparisons (alpha = 0.05 / 4 clusters = 0.0125).

## 3. Results

The mean of the correlations between perceptions of sexism and the beta coefficients are summarized in Table 1. First, we note that the number of subjects is different for each cluster (column *N* in Table 1), due to the rejection of signals with bad quality.

The Wilcoxon signed rank test found a significant negative correlation between perceptions of sexism and beta coefficients was observed in the right cluster (W = 279.0, *p* = 0.001). The correlations were not significant in the other clusters, after Bonferroni correction.

**Figure 2:**
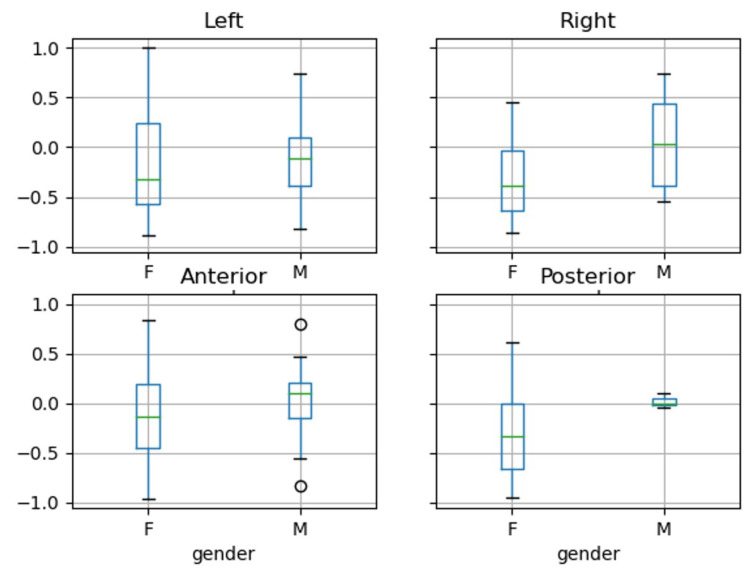
Plot of Spearman’s correlation between perceptions of sexism and beta coefficients by gender for each cluster.

There was also a significant gender difference in the correlation of perceptions of sexism and beta coefficients in the right cluster (Kruskal *p* = 0.005) (Figure 2). This result indicates that the female group showed a significantly stronger negative correlation between perceptions of sexism and brain activation in the right cluster. No other significant differences between genders emerged for the other clusters.

## 4. Discussion

The present study investigated the association between perceptions of sexism and neural activation patterns in response to sexist-related and nonsexist comments. Based on previous studies indicating gender differences in gender stereotype tasks, gender differences in the neural activation patterns were also examined.

A significant negative correlation between perceptions of sexism and neural activation in the right cluster was observed. The right cluster includes the dlPFC and parts of the inferior frontal gyrus, encompassing BA08, BA09, BA45, BA46 and BA47. The results support the first hypothesis that lower dlPFC activity is expected to be observed with higher perceptions of sexism. We propose that this may be similar to lower dlPFC activity observed in response to maternal criticism [11] since both criticism and sexism involve negative emotional reactions. Decreased activity in cognitive control areas towards criticism has been proposed to be associated with detachment from criticism by minimising cognitive processing due to the knowledge that this criticism is hurtful [11]. Experiences of sexism such as stereotypes and derogatory comments or behaviours is commonly met with anger and upset [3] and other such negative emotions including feelings of anger, weakness, tenseness, sadness [7,8,9,10]. Hence, it could be that the more participants perceived the comments to be sexist, the more likely they were to disengage from the contents of the comments, resulting in the lower dlPFC activity observed in response to self-referential sexist-related comments. In addition, previous research has implicated regions in the inferior frontal gyrus in neural models of the perception of semantics and emotions. Specifically, the pars orbitalis division of the inferior frontal gyrus has been demonstrated to participate in the perception of both semantic and emotional content (e.g., [85,86,87]), and has been suggested as an integration zone of semantic and emotional communication where it has a role in the evaluation and action upon communicative signals. Hence, the negative correlation between perceptions of sexism and brain activation in this area may also reflect reduced activity relating to both semantic content perception and processing as well as emotional processing of the sexist-related content.

Another possible explanation for the observed result in the right cluster is the role of right frontal activity in stereotype application and response inhibition. The role of the right IFG in domain-general response inhibition has been proposed to be reflective of efforts towards the inhibition of the influence of stereotypes on behaviour during stereotype judgment tasks [57]. The reduced activity in the right cluster may be reflective of reduced response inhibition, leading participants to apply gender stereotypes and perceive the content as sexist. As such, it could be that the lower the activation in the right cluster, the more participants perceived the content to be sexist due to the application of gender stereotypes in processing the content.

A significant gender difference in the correlation between perceptions of sexism and brain activation also emerged, which supported our hypothesis. This aligns with previous studies indicating greater gender stereotypical bias in males (e.g., [66,72]). The results indicate that there was a stronger negative correlation in the female group compared to the male group, suggesting that greater perceptions of sexism were associated with reduced activity in the right cluster for the female group but not for the male group. In line with the discussion above, the significant gender difference may be reflective of either a gender difference in the emotional response or gender stereotyping. Firstly, the extent of the emotional response and the emotion regulation strategy employed may differ between genders in response to sexism. This may be due to experiences of sexism since females are more likely to be targets of sexist acts compared to their male counterparts in their everyday lives [88]. In addition, previous findings from [30] have suggested that males may be more efficient in terms of neural activity during negative emotion regulation as less prefrontal activity during regulation was observed in males. Hence, it may be that even with greater perceptions of sexism and more negative emotional responses, male participants did not show significant changes in the right cluster to downregulate their negative emotions. Secondly, it is possible that female participants have a greater tendency of response inhibition towards application of gender stereotypes and greater perceptions of sexism were related to reduced response inhibition with the reduced activation in the right inferior frontal gyrus. Conversely, male participants may have a lower tendency of response inhibition in applying gender stereotypes, as reflected by previous results indicating more automatic beliefs about gender stereotypes in males [66], suggesting that men may be more prejudicial.

In summary, the findings in the present study provide initial evidence on the association between perceptions of sexism and neural processing of sexist-related content. Findings indicate that perceptions of sexism may be associated with activity in brain areas associated with emotional and semantic processing as well as response inhibition. The gender difference in this association suggests that there may be differences in the neural processing of self-referential gender stereotypes and sexism, which could be related to findings of differences in the relationship between perceived discrimination and psychological well-being in men and women [89,90]. Future studies can examine neural processing of other sexist incidents such as sexist humour, especially since few studies have looked into the experience of sexism in men and compared these experiences between men and women.

### Limitations

Firstly, the vignettes in the present study were not an exhaustive representation of gender stereotypes and perception of gender stereotypes may differ across individuals. Cultural differences in the perceptions of sexism also imply that future studies can examine neural processing and responses to sexism across different cultural contexts. For example, the same behaviours may not be recognised as sexual harassment by all women [91,92]. Perceptions of the behaviours that constitute sexual harassment have been argued to be rooted in cultural contexts [93,94]. For example, a study among ethnic Chinese, Malays and Indians in Malaysia found that Chinese participants scored significantly lower on perceptions of sexual harassment [95]. Hence, individual differences in sexism and gender roles can also be investigated to understand their influence on perceptions of sexism and the accompanying neural processes.

Secondly, the present study made use of the fNIRS and was only able to image cortical structures. Previous studies have observed co-activation between the pars orbitalis division of the inferior frontal gyrus and subcortical structures such as the ipsilateral amygdala [87]. Given that the amygdala and other components of the core emotion network are subcortical structures, future studies can use functional magnetic resonance imaging (fMRI) to examine the activation patterns of these areas in the context of experiences of sexism as well.

## 5. Conclusions

Although the experience of sexism is common and gender stereotypes about traits and behaviours are pervasive, how individuals perceive and respond to incidents of sexism is not well understood. The findings in the present study provide initial evidence on the association between perceptions of sexism and neural processing of sexist-related content, suggesting that the perception of self-referential gender stereotypes as sexist may be associated with activity in brain areas associated with emotional and semantic processing as well as response inhibition. The gender difference in this association suggests that there may be differences in the neural processing of self-referential gender stereotypes and sexism, implying that experiences of sexism may be perceived and experienced differently between men and women, possibly affecting both groups differently.

## Figures and Tables

**Table 1:** Table of Spearman correlation coefficients by cluster.

		*N*	Spearman’s r	W	*p*
Anterior	Female	36	−0.114 (0.469)	551.5	0.149
	Male	16	0.034 (0.391)		
Left	Female	32	−0.188 (0.527)	335.5	0.013
	Male	16	−0.085 (0.399)		
Posterior	Female	15	−0.307 (0.476)	23.0	0.019
	Male	3	0.022 (0.074)		
Right	Female	34	−0.327 (0.399)	279.0	**0.001**
	Male	16	0.042 (0.433)		

Note. Significant *p*-values are in bold.

## Data Availability

The dataset analysed in this study is available in the open access institutional data repository at the link: https://doi.org/10.21979/N9/C3JRXS (accessed on 18 January 2022).
